# Evaluation of FOXCUT, CCAT2, and HULC LncRNA Expression Levels and Apoptosis Induction by Sodium Butyrate in PC-3 and LNCAP Prostate Cancer Cell Lines

**DOI:** 10.22088/IJMCM.BUMS.10.3.189

**Published:** 2022-01-10

**Authors:** Sanaz Kavousi, Seyed Ataollah Sadat Shandiz, Nastaran Asghari Moghaddam

**Affiliations:** *Department of Biology, Central Tehran Branch, Islamic Azad University, Tehran, Iran.*

**Keywords:** Prostatic neoplasms, RNA, long noncoding RNA, apoptosis

## Abstract

Sodium butyrate (NaBu) is a short-chain fatty acid acting as a histone deacetylase inhibitor, and has been shown to be a potential regulator of cancer cell death. This study aimed to evaluate the effect of NaBu on cell cycle control, apoptosis, and expression of some lncRNAs in two human prostate cancer cells (PC-3 and LNCAP). Cell viability was assessed and the appropriate dose was determined using the MTT assay. Real-time PCR technique was also used to evaluate the expression levels of* HULC, FOXCUT, *and* CCAT2 *lncRNAs. Apoptosis was diagnosed using annexin V staining, and cell cycle distribution was then assessed using flow cytometry with propidium iodide DNA staining. NaBu induced apoptosis in both prostate cancer cell lines in a dose-dependent manner. The expressions of *CCAT2* and *HULC *lncRNAs genes have significantly decreased in the presence of NaBu (P <0.05) in both PC3 and LNCAP cell lines, in comparison with the control. However, no significant difference was observed in the expression of *FOXCUT *lncRNAs. Moreover, the results of flow cytometry showed an increase in cell cycle arrest of LNCAP cell line at the sub-G1 stage as compared to the control cells, but no significant difference was observed between the control cells and NaBu-exposed PC-3 cells. In addition, the percentages of early and late apoptotic cells following treatment with NaBu were 80% and 49.63% in LNCAP and PC-3 cells, respectively. Our results suggest that NaBu has a positive effect on the induction of apoptosis and inhibition of cell cycle in PC-3 and LNCAP prostate cancer cells.

Prostate cancer is the second most common cancer diagnosed in men, which has been ranked as the fifth leading cause of death worldwide with 1,276,106 new cases and 358,989 deaths in 2018. Outbreaks and deaths caused by prostate cancer age increasing worldwide are related to the mean age at the time of diagnosis as 66 years old. By 2040, it is estimated that 2,293,818 new cases will be diagnosed, with a 1.05% increase in mortality (1). Radiotherapy, and ablation hormone therapy, which have largely failed to have positive clinical results, and approximately 30% of the treated prostate cancer patients eventually relapse with the increased specific antigen levels. The advanced prostate cancer is still incurable, and treatment for these patients is only an average survival of 2 years with no progression (2, 3). Therefore, finding new drug therapies is very important in this regard. Sodium butyrate (NaBu) is a short-chain fatty acid that competitively binds to zinc sites of classes 1 and 2 histone deacetylases (HDACs). Accordingly, this binding can conse-quently affect histone acetylation, which ultimately creates a modified DNA structure that alters chromatin. Therefore, it enhances the chromatin's ability to access transcriptional regulatory complexes, leading to the increased transcriptional activation of various genes. Butyrate is a HDAC inhibitor, which stops the cell cycle at the G1 or G2/M stages, and increases the expressions of other genes and proteins involved in cell differentiation and apoptotic signaling (4-6). Long non-c0ding RNAs (lncRNAs) have a size range from about 200 nucleotides to less than 100 kb (7). These lncRNAs are transcribed by RNA polymerase from one of the DNA strands, and may be present within the nucleus or cytoplasm of the cells (8). LncRNAs and non-coding RNAs are effective regulators of gene expression. Changes in the expression of these RNAs play a role either in reducing or increasing the expressions of their target genes. The functional properties of highly up-regulated in liver cancer (*HULC*) lncRNA have shown that it induces tumor phenotypes such as cell viability, proliferation, invasion *in vitro*, as well as tumor growth and angiogenesis *in vivo*. Studies show that impaired *HULC* regulation plays a major role in tumorigenesis (9). *FOXC1* upstream transcript (*FOXCUT*) lncRNA is critical for a wide range of tumor developmental and homeostatic processes, including proliferation, differentiation, apoptosis, metastasis, and invasion (10).

It is possible that NaBu is not only a prophylactic agent in the large intestine of people that typically consumes high-fiber diets, but it may also act as a therapeutic agent (11-13). In recent years, changes in gene expression have been extensively studied in people with cancer. In this study, the transcriptional expression levels of three lncRNAs namely colon cancer associated transcript 2 (*CCAT2*), *HULC*, and *FOXCUT*, and the effect of NaBu on cell cycle control, apoptosis, and expressions of these lncRNAs were evaluated in two prostate cancer cell lines, PC-3 and LNCAP.

## Materials and methods

**Eukaryotic cell culture and conditions**

Two prostate cancer cell lines (PC-3 and LNCAP) were obtained from the Pasteur Institute Cell Bank of Tehran, and were revived in sterile flasks (Nunc, Denmark) with complete Dulbecco’s Modified Eagle’s Medium (DMEM) (Gibco, Scotland) culture medium containing 10% FBS (Thermo Fisher Scientific, USA), and 1% streptomycin antibiotic (Gibco, Scotland), which were then incubated in a humidified cell culture incubator with 5% CO_2_ at 37 °C.

**Measurement of vital cell activity by MTT method**

Growing PC-3 and LNCAP cells (10000 cells per well) were cultured in 96-well plates. Thereafter, they were treated with NaBu) CAS Number 156-54-7; Product Number: S0519 Sigma-Aldrich, Germany) in various concentrations (0.5, 1, 2.5, and 5 mM) for 24, 48, and 72 h. The supernatant of the cells was then removed. Subsequently, 100 μl MTT solution (5 mg/ml) was added to each well. Next, the LNCAP and PC-3 cells were treated overnight with MTT solution at 37 °C. Finally, DMSO solvent was used to dissolve the formazan crystal. After 2 h, the adsorption of the samples was read using ELISA reader (Eppendorf, Germany) at 570 nm. Doxorubicin was considered as the positive control group. The percentage of living cells was calculated using the following formula: 

% cell viability = [OD sample/OD control] ×100

The concentration of IC_50_ was estimated using Prism graph pad 6 software version 25 (14).

**Detection of apoptotic cells by flow cytometry**

Cell apoptosis was assessed by annexin V staining kit (IQ Product, Germany) followed by the flow cytometry analysis. The PC-3 and LNCAP cell lines were poured into 6 cell plates for 24 h; 2.5 mM NaBu was then added, and after 48 h the cells were trypsinized, centrifuged, and afterwards, annexin V was incubated for 15 min at room temperature for evaluation by flow cytometry (15).

**Investigation of the expression levels of** ***HULC*** **,** ***FOXCUT*** **, and** ***CCAT2*** **lncRNAs**

Levels of *HULC, FOXCUT, *and* CCAT2 *lncRNAs expression in PC-3 and LNCAP cell lines of prostate cancer were measured using real time PCR method. Total cellular RNA was isolated from these cells in terms of the manufacturer's protocol (Roch, Germany) using Trizol. According to the instructions proposed in the Qiagen kit (Germany), 1 μL total RNA was reversed to the transcriptional cDNA. Afterward, qRT- PCR was performed using the SYBR Green dye in the PCR detection system. The *GAPDH* was considered as the internal control gene. The primers utilized for qRT- PCR are listed in Table 1. Finally, the relative expression level of the genes was calculated using 2^-∆∆ct^ method. 

**Investigation of cell cycle inhibition**

Firstly, the PC-3 and LNCAP cells (500,000 cells per well) were poured into each well in 6 plates, and after 24 h the supernatant culture medium of the cells was completely emptied, treated with fresh whole culture medium containing 2.5 mM NaBu, and then incubated for 5 h. Subsequently, the cells were washed with PBS and trypsin, isolated from the bottom of the plate and after re-washing with PBS, they were prepared for flow cytometry (16). Distribution in different phases of the cell cycle called sub-G1, G0 / G1, G2 / M, and S was also examined. 

**Statistical analysis**

Statistical significance between treatment and control groups were obtained using two-way analysis of variance and the Turkey’s tests (SPSS Statistics software, version 22). Data were presented as the mean ± standard deviation of three replicates from three independent experiments.

**Investigation of cell viability based on colori-metric MTT assay**

In this study, MTT method was used to compare the number of living cells in the treated groups with different doses of NaBu at different times compared to the control groups. In this phase, Doxorubicin was considered as the positive control group. Based on the obtained results, there was a significant difference in the PC-3 cell line at 5 and 2.5 mM NaBu and doxorubicin concentrations at 24, 48, and 72 h in comparison with the control.

## Results

**Table 1:** Sequence of primers for *GAPDH, HULC, FOXCUT, *and* CCAT2 *lncRNAs genes

**Sequence (** **5´ → 3´)**	**Forward (F)** **and Reverse (R)**	**Genes**
5´ GTGGTCTCCTCTGACTTCAAC3´	F	*GAPDH*
5´ GGAAATGAGCTTGACAAAGTGG3	R	
5´ ACAGACCAAAGCATCAAGCA3´	F	*HULC*
5´TTTGCCACAGGTTGAACACTT3´	R	
5´ GCAAGTCCCAACAAGAAGC3´	F	*CCAT2*
5´ ACTGAAATGGTGCTGCTGGT3´	R	
5´ GGCGGGAACGATCAGAAAT3	F	*FOXCUT*
5´ CGACCTTGGGCAGATACTCC3	R	

group (P <0.05). At 1 mM, there was a significant difference with the control group after 24 and 72 h of exposure, while this difference was not significant after 48 h (Figure 1). NaBu-exposed PC-3 cells for all the time intervals at 0.5 mM concentration did not significantly increase the cytotoxicity in comparison with the control groups (Figure 1A). Figure 1B indicated the cell viability of LNCAP that revealed a significant cytotoxic activity at any concentration after 72 h exposure to NaBu. Comparison of the means in cell lines were also performed using the two-way ANOVA. **Results of gene expression using qRT-PCR**

In the present study, the quantitative real time PCR technique was used to analyze the genes expression data. Genes expression in the control cells and treated groups was evaluated by 2.5 mM NaBu after 48 h. To measure the level of gene expression in different groups, the expression of *GAPDH* was considered as a control for normalizing changes in gene expression. As shown in figure 2, the expressions of *CCAT2* and *HULC *lncRNAs have decreased significantly in both PC3 and LNCAP cell lines treated with NaBu (P <0.05). Notably, there was no significant change in *FOXCUT lncRNAs* gene expression in the treated samples in comparison with the controls.

**Fig.1:**
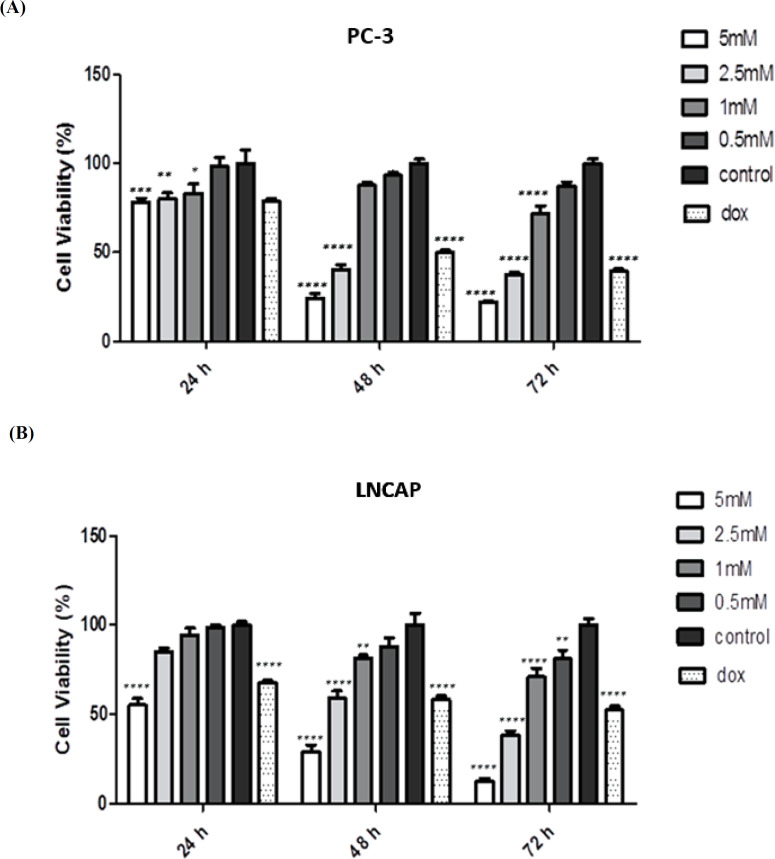
**Comparison of FOXCUT, CCAT2, HULC lncRNAs gene expression in prostate cancer cells.** A) PC3 cells; B) LNCAP cells. Cells were treated with 2.5 mM sodium butyrate nanoparticles and genes expression were assessed in comparison with control group (P < 0.05).

**Cell cycle inhibition**

To evaluate the effect of NaBu on the cell cycle distribution, propidium iodide (PI) staining was used with flow cytometry. In case of any apoptosis and cell membrane damage, the dye is able to pass through the membrane. The amount of DNA content indicate different phases of the cell cycle. The percentage of cell population was shown in different phases. According to the results of cell cycle inhibition in the LNCAP cell line of the treated group (Figure 3A), the frequencies of cells at different stages of the cell cycle were as follows: G1: 62.15%, S:10.13%, G2: 4.38%, and sub-G1: 24.12%. Sub-G1 ratio is expected to increase in the treatment group in comparison with the control group. The results of cell cycle inhibition at different stages of cell cycle for the LNCAP cell line control group (Figure 3B) are as follows: G1: 60.22%, S: 30.11%, G2: 14.93%, and sub-G1: 0.35%. 

Results of cell cycle inhibition in the PC-3 cell line treated group (Figure 4A) are as follows: G1: 80.01%, S: 10.97%, G2: 9.12%, and sub-G1: 2.95% versus G1: 65.5%, S: 24.51%, G2: 15.2%, and sub-G1: 0.53% in control cells, indicating an increase of sub-G1 population in NaBu-exposed PC-3 cells; however, no significant change was observed in the treated group as compared to the control group. 

**Fig.2:**
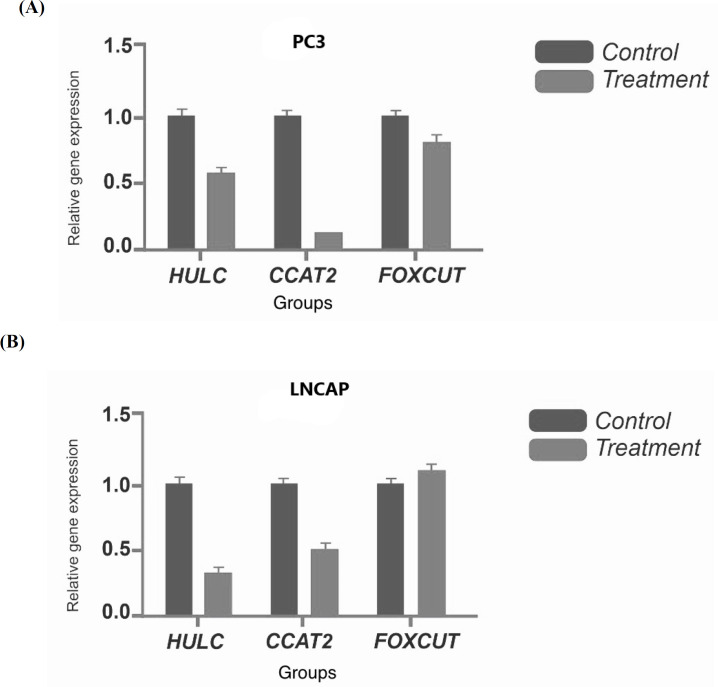
**Comparison of FOXCUT, CCAT2, HULC lncRNAs gene expression in prostate cancer cells.** A) PC3 cells; B) LNCAP cells. Cells were treated with 2.5 mM sodium butyrate nanoparticles and genes expression were assessed in comparison with control group (P < 0.05)

**Fig.3:**
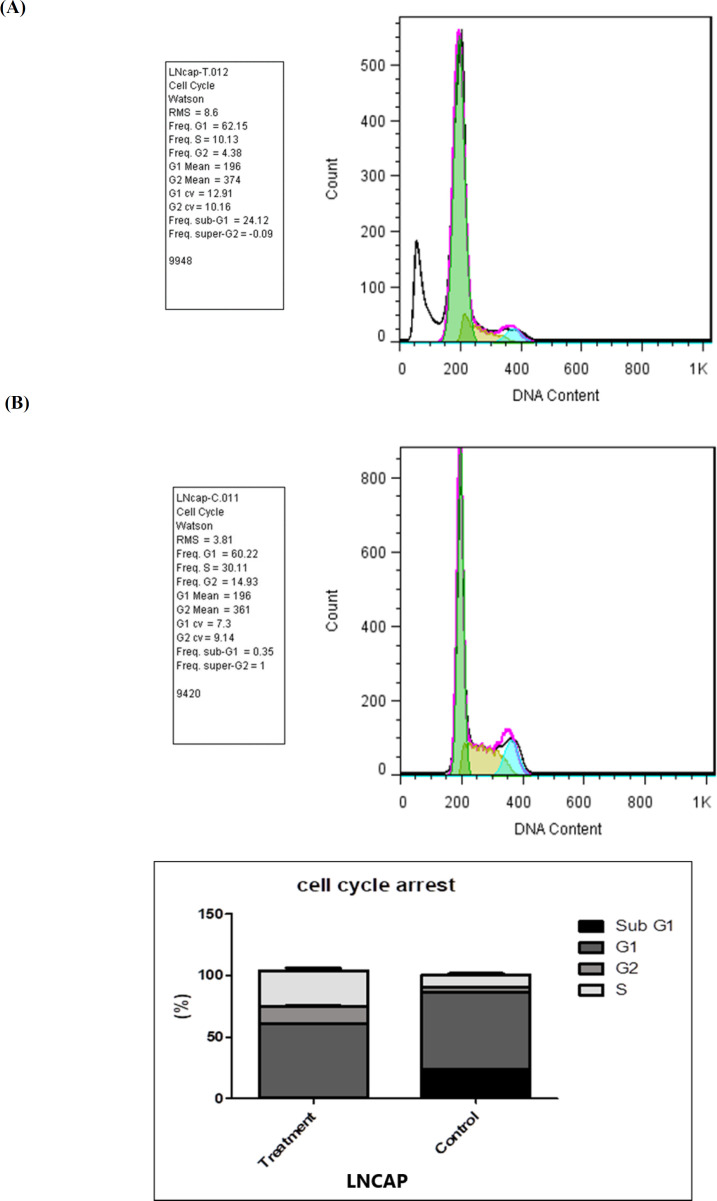
**Assessment of cell cycle inhibition.** A) LNCAP cells treated with sodium butyrate at a dose of 2.5 mM; B) Non-treated LNCAP cells (control sample)

**Fig.4:**
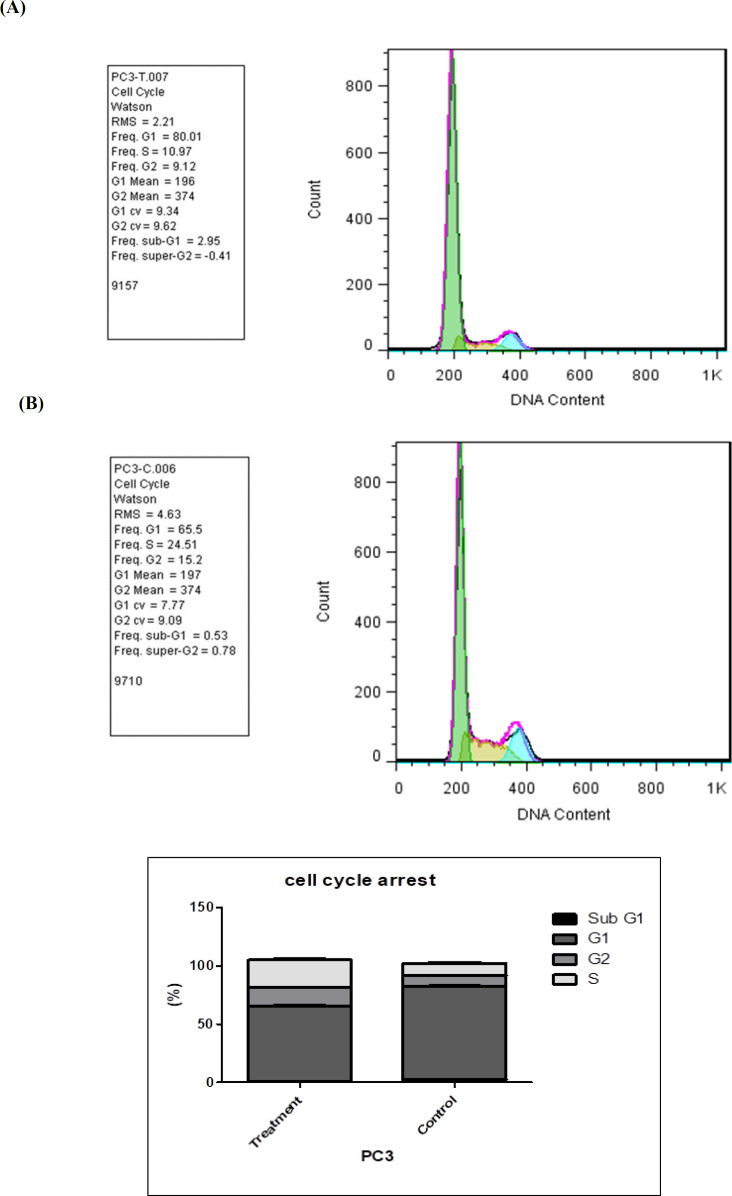
**Assessment of cell cycle inhibition.** A) PC3 cells treated with sodium butyrate at a dose of 2.5 mM; B) Non-treated PC3 cells (control sample).

**Fig.5:**
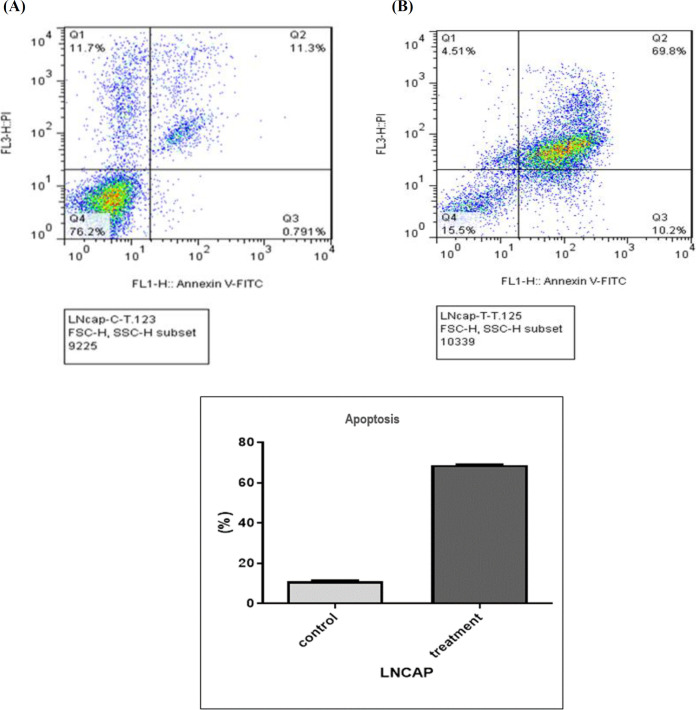
**Flow cytometry results of apoptosis by annexin V staining.** A) Sodium butyrate-treated LNCAP cells at a dose of 2.5 mM; B) Non-treated LNCAP cells (control sample)

**Apoptosis evaluation by flow cytometry**

Annexin V is used in flow cytometry to detect apoptotic cells through its ability to bind to phosphatidyl serine, which is known as a marker of apoptosis. During apoptosis process, phosphatidyl serine travels from the inner membrane to the cell surface and then bonds with annexin V. As shown in figures 5 and 6, Q1 indicates necrosis, Q2 delayed apoptosis, Q3 early apoptosis, and Q4 indicates viable cells. Moreover, the percentage of late apoptosis in NaBu-treated LNCAP cells was 69.8%, while the percentage of the control cells was 11.3%. Additionally, the percentage of apoptosis in PC3 cells treated with NaBu was 39.9% and 2.21% in the control PC3 cells.

## Discussion

Excessive cell proliferation along with apoptotic attenuation and cell removal are considered as the prominent features of different types of cancer. Accordingly, the induction of apoptosis is known as an efficient molecular approach to improve the balance of cell death when reducing apoptosis (6). NaBu is a compound that has a variety of effects on the cultured mammalian cells, including multiplicative skills, inducing differentiation, and gene regulation. Therefore, it can be used in *in vitro* studies (11, 17). Transcription of HDAC inhibitor genes has led to apoptosis in cancer cells through new promising therapies for the treatment of various types of human cancer. Altogether, these results suggest that NaBu may have the potential to act as a new class of low-toxic anticancer drug. However, the mechanisms of action of these drugs are not fully understood yet (6, 18-20). According to the studies performed by Shin et al., NaBu is able to activate various neurocellular anti-cancer mechanisms, including cell cycle arrest, cell differentiation, and activation of HDAC inhibitor genes . Transcription of HDAC inhibitor genes has led to apoptosis in cancer cells through new promising therapies for the treatment of various types of human cancers (6). According to a study conducted by Wang *et al*. (2020), NaBu is able to inhibit cell proliferation and stop the cell cycle in G2/M, S or G1 according to the type of cancer cells (21). In this study, we investigated the effect of NaBu on the expressions of* FOXCUT*, *HULC, *and* CCAT2* lncRNAs in two prostate cancers; PC-3 and LNCAP cell lines. By comparing the data of cell viability in groups treated with different doses at different times , a difference was found in the mean number of living cells in both LNCAP and PC-3 cell lines. In this study, doxorubicin was considered as the positive control. In fact, these results show the effect of drug dose and test duration on the biological ability of cells, meaning that there is a significant difference between the two doses of 5 and 2.5 mM at 48 and 72 h with the control group and also after 24 h. Cell viability was assessed at a dose of 2.5 mM to be continued by MTT assay. In addition, the results of real time PCR in PC-3 and LNCAP cells showed that the expressions of *HULC *and *CCAT2* lncRNA genes were significantly different in the treated and control groups, as they were downregulated in treated groups, but no difference was observed between these groups in terms of *FOXCUT *lncRNA gene. Flow cytometry results of apoptosis using annexin V showed a higher percentage of apoptosis in LNCAP and PC-3 cells treated with NaBu compared to the control sample. In a similar study by Semaan *et al*. in 2020 showed the effect of NaBu dose and test duration on the rate of apoptosis in the MCF-7 cell line, and also the results of flow cytometry revealed the cell cycle arrest in G1 phase (22). Another similar study by Amiri *et al*. reported the effect of NaBu on growth inhibition and apoptosis induction in the colon cancer HT-29 cell line, which can be due to the increased *BID* gene expression, and apoptotic gene expression was shown to be altered by histone deacetylase inhibitors (23). Correspondingly, both studies are in line with our study; showing the effect of NaBu on apoptosis induction and growth inhibition in cell lines. Moreover, they confirm the effect of drug dose and test duration on the rate of apoptosis induction.

In addition, the results of cell cycle inhibition confirmed the apoptosis of cells, which were treated at the same time with the same concentration and under the same conditions relative to the control sample. A review conducted on various studies, including a study conducted by Shin et al., revealed that NaBu can be considered as a new drug for chemotherapy. Moreover, as an agent, it is able to activate various anti-cancer mechanisms including cell cycle arrest and cell differentiation (6).

In our study, the increased percentage of cells at the sub-G1 stage in the treatment group compared to the control group confirmed the effect of NaBu on increasing apoptosis.

Additionally, in a study by Salimi *et al*., it was reported that NaBu reduces the viability of breast cancer cells depending on the dose and time. Besides, in this study, the appropriate range of NaBu concentration was examined at different time intervals. Finally, their results showed that the cytotoxic effect of NaBu is related to the apoptosis induction in both cell lines; however, the rate of apoptosis induction of NaBu was significantly lower in MCF-10A compared to breast cancer cells (24). Natoni et al. have shown that NaBu can also sensitize human pancreatic cancer cell lines by both intrinsic and extrinsic ways of apoptosis. Furthermore, it was found that NaBu can increase the level of intracellular reactive oxygen species (ROS) production in some cell lines, but not in others. ROS accumulation has also been shown to impair some cellular functions and promote apoptosis (25). The results of our study are consistent with those of previous studies; confirming the positive effect of NaBu on increasing the induction of apoptosis and cell cycle inhibition in cancer cells. Increasing the level of *CCAT2 *can induce chromosomal instability, which ultimately leads to the increased number of ploid cells (26, 27). *HULC *can also increase the proliferation of hepatocellular carcinoma cells (28).

The results of lncRNAs expression showed that there was a significant difference between the control and treated groups in *CCAT2* and *HULC *lncRNAs in both cancer cell lines. It was demonstrated that the regulation of *FOXCUT* expression levels was associated with poor differentiation, lymph node metastasis, and a worse prognosis. Silent *FOXCUT* could also induce cell proliferation, invasion, migration, colony formation, and *FOXC1 *gene expression. FOXC1 and FOXC2, some FOX subfamilies, appear to play a new role in cancer progression (10). FOXC1 also acts as a downstream target for growth factor-converting β (TGF-β) to prevent tumorigenesis. Increased *FOXC1* expression causes cells to be sensitive to TGF-β1-mediated growth inhibitory effects and stop cells in the G0/G1 phase (18). Here, we evaluated the up regulation of *FOXCUT* in LNCAP cells. However, we have observed no difference between the control and treatment groups in terms of the *FOXCUT* lncRNA expression. 

In conclusion, the results of the data presented here show that NaBu could manipulate the growth of prostate cancer cells and induce apoptosis in two prostate cancers LNCAP and PC-3 cells compared to the control sample. The expressions of *CCAT2* and *HULC *lncRNAs genes in the treated sample with NaBu have significantly decreased in both PC3 and LNCAP cell lines, which had significant differences in their expressions compared to the control sample. However, no significant difference was observed in the expression of *FOXCUT *lncRNAs. The increased percentage of cells at the sub-G1 stage in the treatment group compared to the control group confirmed the effect of NaBu on increasing apoptosis. Our data confirmed the positive effect of NaBu on the apoptosis induction in prostate cancer cells.

## Conflict of Interest

The authors declare that there is no conflict of interest regarding the publication of this manuscript.
